# South–West of England’s Experience of the Safety and Tolerability Pirfenidone and Nintedanib for the Treatment of Idiopathic Pulmonary Fibrosis (IPF)

**DOI:** 10.3389/fphar.2018.01480

**Published:** 2018-12-17

**Authors:** Shaney L. Barratt, Sarah Mulholland, Khaled Al Jbour, Henry Steer, Markus Gutsche, Noeleen Foley, Rajiv Srivastava, Charles Sharp, Huzaifa I. Adamali

**Affiliations:** ^1^Bristol Interstitial Lung Disease Service, North Bristol NHS Trust, Bristol, United Kingdom; ^2^Gloucestershire and Cheltenham General Hospitals, Gloucestershire Hospitals NHS Foundation Trust, Gloucester, United Kingdom; ^3^Royal United Hospitals Bath NHS Foundation Trust, Bath, United Kingdom; ^4^Weston General Hospital, Weston-super-Mare, United Kingdom

**Keywords:** idiopathic pulmonary fibrosis, anti-fibrotic medications, nintedanib, pirfenidone, interstitial lung disease

## Abstract

**Purpose:** Pirfenidone and nintedanib are two novel antifibrotic agents licensed for the treatment IPF. Prior to being approved for use in England for patients with FVC >50% and <80%, these were made available for all IPF patients under the Mild Patient Program (MPP) and Patient In Need Scheme (PIN). Prescribing of these medications is restricted to specialist centers. We sought to characterize the population of patients prescribed antifibrotics and determine the drug tolerability of these medications in the Northern hub of the Southwest of England regional ILD network.

**Methods:** A retrospective analysis of all patients treated with antifibrotics between August 2012 and July 2017 was undertaken. Baseline characteristics including patient demographics and pulmonary physiology, in addition to drug tolerability and reasons for treatment cessation were collated. Data were compared using unpaired student’s *t*-test, Chi-squared, Mann–Whitney rank sum or ANOVA as appropriate. Logistic regression analysis evaluated clinical characteristics associated with discontinuation of pirfenidone therapy. *P* < 0.05 was considered statistically significant.

**Findings:** A total of 164 patients, all with consensus diagnoses of IPF, were identified. Of these, 70.1% (115/164) received pirfenidone as their initial therapy. Baseline age, gender and pulmonary physiology did not differ significantly between groups. Drug discontinuation occurred most commonly due to adverse drug reactions events (ADRs) for both pirfenidone [40.0% (46/115)] and nintedanib [16.3% (8/49)]. Anorexia, rash and gastrointestinal disturbance were reported most commonly as the reason for cessation of pirfenidone; anorexia, nausea and weight loss for nintedanib. Duration of therapy prior to discontinuation because of ADRs did not differ significantly between medication groups but patients with a baseline FVC < 65% predicted, had a significantly shorter duration of pirfenidone prior to discontinuation due to ADRs, compared to those with a FVC 65–80% predicted. Multivariate logistic regression did not identify any independent baseline characteristics that predicted discontinuation of pirfenidone therapy prior to 52 weeks.

**Implications:** Idiopathic pulmonary fibrosis (IPF) patients treated with nintedanib had comparable treatment emergent adverse event (TEAE) profiles in clinical practice to those reported in clinical trials. The TEAE profile of pirfenidone was higher than clinical trial data would suggest, although comparable to real-world datasets. Further work is required to explore the possible reasons underpinning this finding, including whether this is related to population co-morbidities or center threshold. No new safety concerns were identified.

## Introduction

Idiopathic pulmonary fibrosis (IPF) is a progressive fibrotic lung disease which ultimately leads to inexorable decline in lung function, culminating in respiratory failure and death with median survival of 2–5 years from diagnosis (Raghu et al., 2018).

The Southwest region of England covers a large geographical area, occupying approximately 18% of England. It has a population of approximately 4.6 million, accounting for 8.6% of the United Kingdom’s total population (Office for National Statistics, 2017). There are 14 acute hospitals and 11 clinical commissioning groups (CCGs) within the region. To unify the availability of services across the region, a hub and spoke model of care has been developed, with two specialist interstitial lung disease (ILD) centers (hubs) dividing the North and the South of the Southwest, each working alongside their affiliated hospitals (spokes).

Two anti-fibrotic therapies are currently approved for the treatment of IPF. Pending approval from the National Institute for Health and Clinical Excellence (NICE) institution in England, both medications were made available to patients by manufacturers under the remit of the mild patient program (MPP) for pirfenidone and the individual patient supply program (IPSP) and the patient in need (PIN) scheme for nintedanib. Under current NICE guidance, antifibrotic therapy can be recommended as an option for treating IPF if the patient has a FVC (Forced Vital Capacity) of between 50 and 80% predicted. Treatment should be stopped if there is evidence of disease progression (an absolute decline of 10% or more in predicted FVC within any 12 months period) (National Institute for Health and Care Excellence, 2013).

Pirfenidone became the first European licensed therapy for IPF in 2011 (Annex, 2014) with the simultaneous FDA (Food and Drug Administration) approval of pirfenidone and nintedanib in 2014 (Raghu and Selman, 2015). Pirfenidone is a novel drug demonstrating anti-inflammatory and anti-fibrotic properties, although the precise mechanism of action in IPF remains unknown (Kaneko et al., 1998; Oku et al., 2008; Schaefer et al., 2011). A significant reduction in FVC decline in IPF patients treated with pirfenidone was demonstrated in one of two concurrently run international CAPACITY (Clinical Studies Assessing Pirfenidone in Idiopathic Pulmonary Fibrosis: Research on Efficacy and Safety Outcomes) (Noble et al., 2011) randomized controlled trials (RCTs). A further phase III study, ASCEND (Assessment of Pirfenidone to Confirm Efficacy and Safety in IPF) (King et al., 2014), requested by the US FDA (United States Food and Drug Administration) due to the discrepancies in primary endpoints of the CAPACITY trials, demonstrated that Pirfenidone reduced lung function decline with improved progression free survival of IPF patients at 52-weeks. The adverse events most commonly reported in these clinical trials and in the open-label follow-up extension study (RECAP) (Costabel et al., 2017) were gastrointestinal and skin related. The longer-term safety and tolerability profiles (PASSPORT) seem consistent with the initial clinical trial data (Koschel et al., 2014).

Nintedanib was granted marketing authorization for use in the European Union in January 2015 (European Medicine Agency, 2014). It is a tyrosine kinase inhibitor targeting Vascular-endothelial growth factor (VEGF), PDGF and fibroblast growth factor (FGF) receptors. In the phase II TOMORROW (Richeldi et al., 2011) and replicate phase III INPULSIS 1 and 2 trials (Richeldi et al., 2014), nintedanib 150 mg twice daily significantly reduced the decline in FVC of IPF patients at 52-weeks, compared to placebo, and had positive effects on time to first acute exacerbation. Gastro-intestinal related adverse events were reported most frequently. Similar drug safety and tolerability has been reported the open-label extension study of nintedanib (INPULSIS-ON) (Crestani et al., 2016).

We sought to determine the drug tolerability of pirfenidone and nintedanib in our clinical practice within the North hub of the Southwest of England regional ILD network.

## Materials and Methods

This is a retrospective observational study of all IPF patients prescribed anti-fibrotic therapy by the Northern hub (North Bristol NHS Trust) of the Southwest of England regional ILD network: formed in conjunction with Royal United Hospitals Bath NHS Foundation Trust, Gloucestershire Hospitals Foundation Trust and Western General Hospital.

Pirfenidone and nintedanib were given as continuous oral treatments in standard maintenance dosing schedules of pirfenidone 801 mg three times daily and nintedanib 150 mg twice daily.

Baseline characteristics including patient demographics and pulmonary physiology, in addition to information on drug tolerability and reasons for cessation of therapy, were collated for all IPF patients who were prescribed nintedanib or pirfenidone between August 2012 and July 2017. All patients had a multidisciplinary team (MDT) diagnosis of IPF, in accordance with the ATS/ERS/JRS/ALAT consensus guidelines (Raghu et al., 2018).

Data on adverse drug reactions (ADRs), irrespective of causality, were collected from electronically stored transcripts of patient and caregiver communications with our specialist pharmacist or ILD specialist nurses as part of our patient support program or from clinical letters recording each ILD outpatient clinic appointment, written by members of the ILD team (doctors, specialist ILD nurses or pharmacist). Discontinuation of therapy due to disease progression (as evidenced by a decline in percent predicted FVC of 10% or more within any 12 months period) or death of the patient was also determined; together with the ADRs these were collectively termed as treatment emergent adverse events (TEAEs). The study was approved as a service evaluation project by the North Bristol NHS Trust before commencement.

Categorical variables are presented as counts, whilst continuous variables are presented as means ± standard deviation (SD). Unpaired Student’s *t*-test with Welch’s correction, Mann–Whitney rank sum or Chi Squared testing was used for comparison of two groups as appropriate. ANOVA with *post hoc* Holm’s Sidak multiple comparisons analysis was used for comparisons of multiple groups. Kaplan Meier curve analysis was used to determine the median time of follow up prior to discontinuation of therapy. Logistic regression analysis evaluated clinical characteristics associated with discontinuation of pirfenidone therapy. For all tests a *P* < 0.05 was considered statistically significant. Data were analyzed using GraphPad prism software, version 7.0 (GraphPad Software Inc., San Diego, CA, United States) and R version 3.4.4 for logistic regression analysis.

## Results

### Baseline Demographics Pirfenidone and Nintedanib IPF Populations

A total of 167 patients, all with multidisciplinary consensus diagnoses of IPF, were identified as receiving pirfenidone or nintedanib during the study period. Pirfenidone and nintedanib were not used concurrently. Three patients opted out of anti-fibrotic medication after initial authorization paper work had been completed and were thus excluded from the analysis. Of the final cohort of 164 IPF patients, 70.1% (115/164) received pirfenidone as their initial therapy.

**FIGURE 1:**
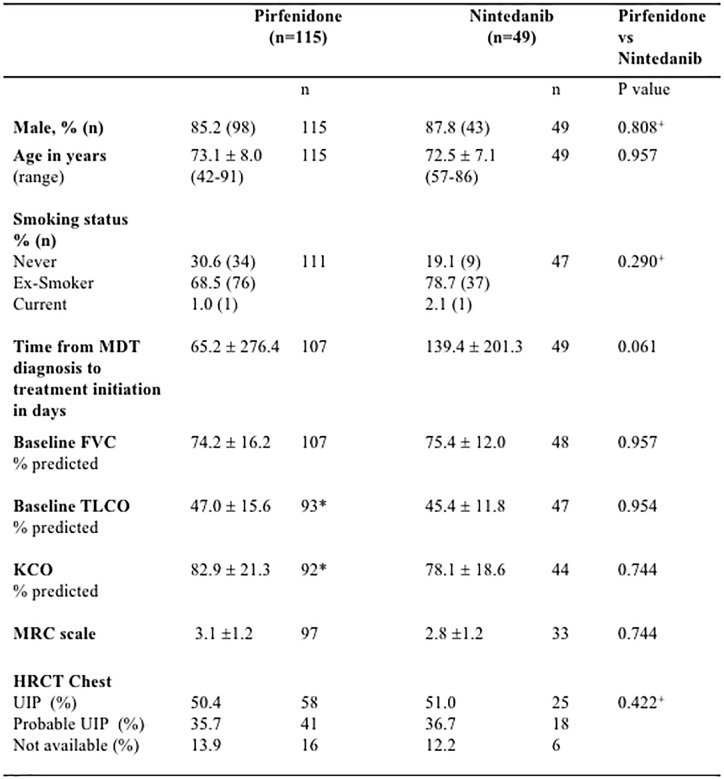
Baseline demographics of Idiopathic Pulmonary Fibrosis (IPF) patients receiving Pirfenidone or Nintedanib. There were no statistically significant differences in baseline demographics between pirfenidone and nintedanib subgroups. n, number; FVC, forced vital capacity; TLCO, transfer factor for carbon monoxide; KCO, transfer coefficient; MRC, medical research council; HRCT, high resolution computed tomography; UIP, usual interstitial pneumonia; SD, standard deviation. *^∗^* 5 patients were unable to perform the gas transfer maneuver due to cough. HRCT definitions of radiological pattern are as defined by the ATS/ERS/JRS/ALAT 2018 guidelines for IPF (Raghu et al., 2018). Data presented as means with standard deviation unless otherwise stated. Statistical significance of continuous variables were analyzed using Holm–Sidak multiple comparisons analysis, without assuming a consistent SD. ^+^ Categorical variables were analyzed using Chi-squared tests. *P* < 0.05 was considered statistically significant.

The majority of patients in pirfenidone and nintedanib treatment cohorts were male [pirfenidone: 85.2% (98/115), nintedanib: 87.8% (43/49)] and ex-smokers [pirfenidone: 68.5% (76/111), nintedanib: 78.7% 37/47]. Pulmonary function was restrictive: mean FVC was 74.4% (SD 16.2) for pirfenidone and 75.4% (SD 12.0) for nintedanib cohorts, with a moderate reduction in TLCO [pirfenidone 47.0% (SD 15.6), nintedanib 45.4% (SD 11.8)]. Baseline age, gender, smoking status, pulmonary physiology, MRC dyspnoea score, time to treatment initiation and radiological pattern identified on HRCT Chest, did not differ significantly between nintedanib and pirfenidone treatment groups (Figure 1).

Patients in the pirfenidone treatment group were significantly older and a higher proportion were male compared to published pooled data of the ASCEND/CAPACITY trials (Noble et al., 2016), but had comparable baseline FVC and TLCO %predicted (Supplementary Figure S1A). Patients in the nintedanib treatment group were comparable to those in the INPULSIS 1+2 trials (Richeldi et al., 2011) in terms of age, gender and baseline FVC and TLCO %predicted (Supplementary Figure S1B).

### Overall Tolerability of Anti-fibrotic Medications

**FIGURE 2:**
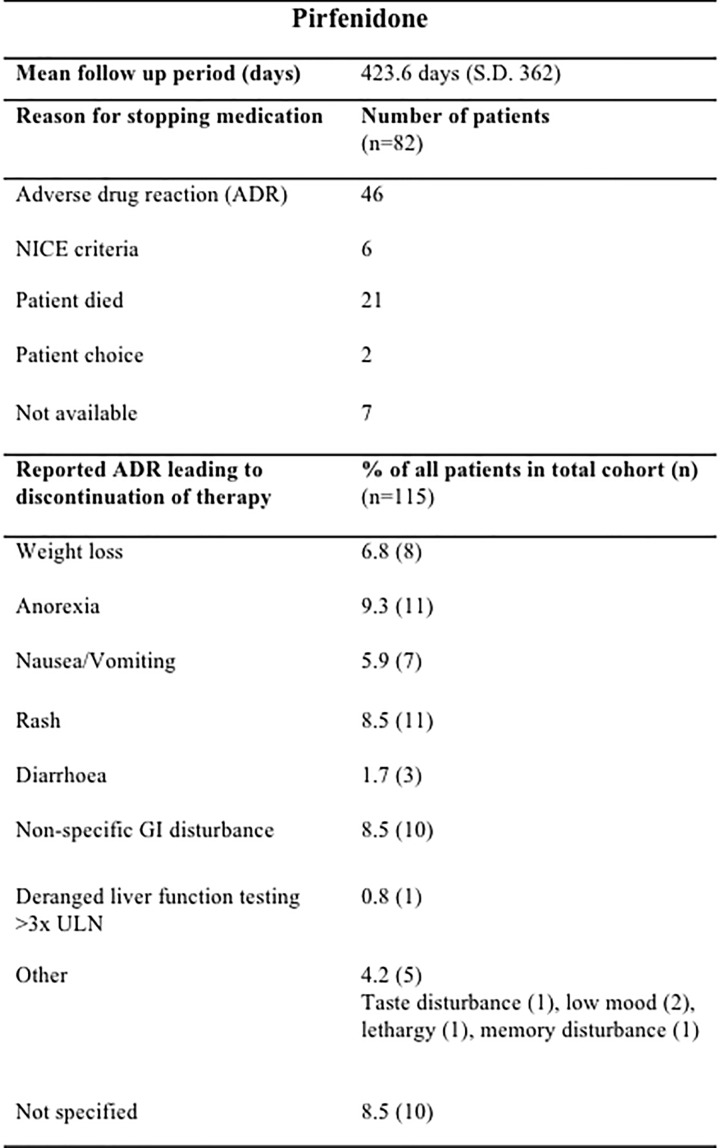
Documented reasons for cessation of Pirfenidone therapy during the study with a mean duration of follow up of 423.6 days (SD 362). In the cohort studied, 82/115 patients terminated therapy, most commonly due to ADRs (46/82), whilst fulfillment of NICE criteria for disease progression [National Institute for Health and Care excellence (NICE) criteria for stopping medication (National Institute for Health and Care Excellence, 2013): anti-fibrotic medication has to be stopped where there is disease progression as evidenced by a decline in percent predicted FVC of 10% or more within any 12 months period] 6/82, patient choice 2/82 and death 21/82 were also documented reasons. Anorexia (15 ADRs), rash (11ADRs) and GI disturbance (10 ADRs) were the most commonly reported ADRs leading to discontinuation of pirfenidone in our cohort. n, number; GI, gastrointestinal; ULN, upper limit of normal; GORD, gastro-oesophageal reflux disease; URTI, upper respiratory tract infection.

**FIGURE 3:**
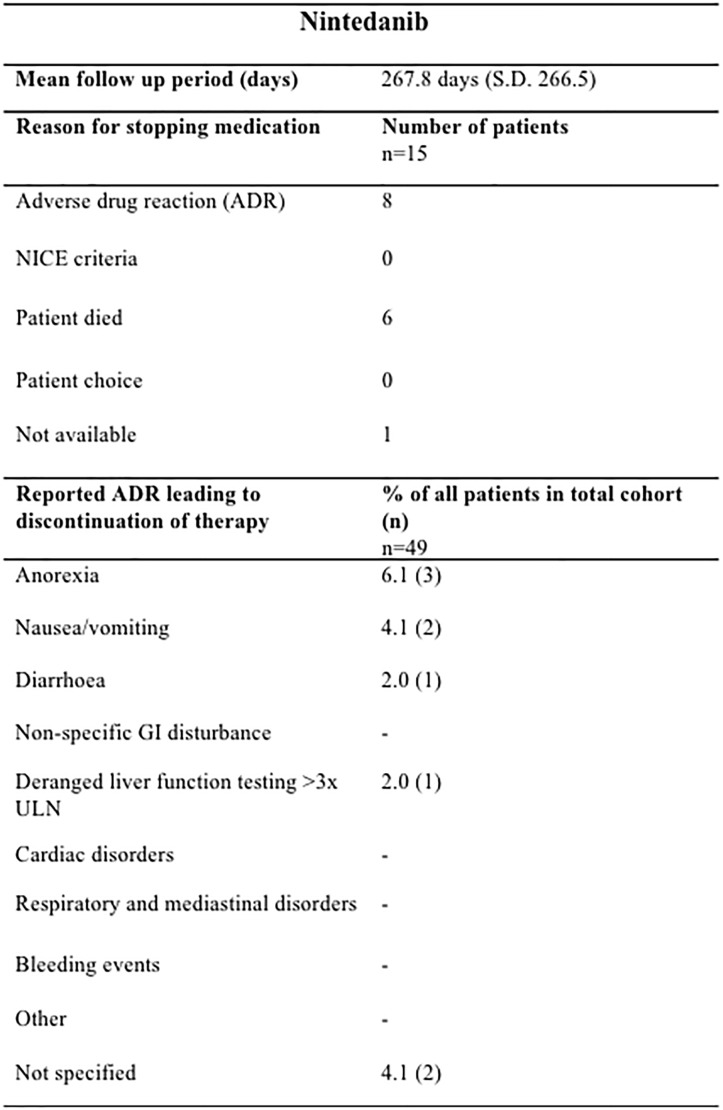
Documented reasons for cessation of Nintedanib therapy during the study with a mean duration of follow up of 267.8 days (SD 266.5). In the cohort studied, 15/49 patients terminated therapy, most commonly due to ADRs (8/15) but also due to patient death (6/15). Anorexia (3 ADRs), nausea (2 ADRs) and weight loss (2 ADRs) were most commonly reported in our cohort. n, number; GI, gastrointestinal; ULN, upper limit of normal.

The mean treatment follow-up period was 423.6 days (SD 362) for pirfenidone and 320.9 days (SD 220.9) for nintedanib. Pirfenidone and nintedanib were discontinued most commonly due to ADRs; 40.0% (46/115) of those prescribed pirfenidone (Figure 2) and 16.3% (8/49) nintedanib (Figure 3).

**FIGURE 4:**
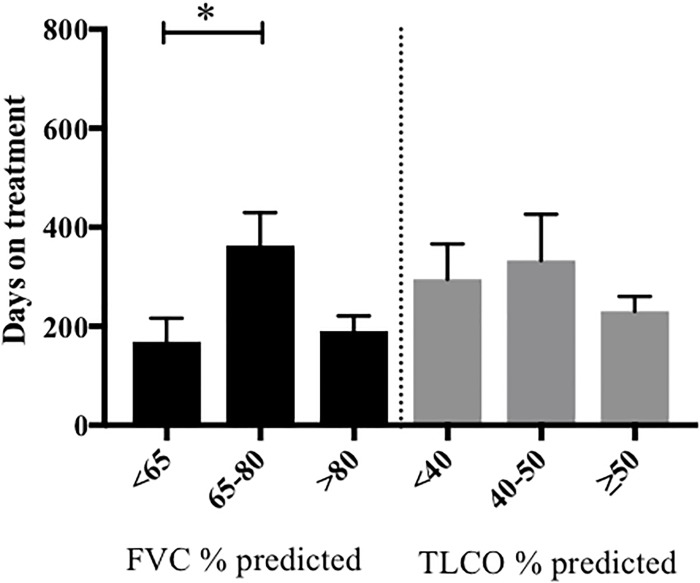
Length of pirfenidone treatment prior to discontinuation due to ADRs, stratified according to baseline pulmonary function. The duration of pirfenidone therapy was examined in patients who had discontinued treatment due to ADRs and was then stratified according to their baseline FVC (% predicted) and TLCO (% predicted). Data was available for 40/46 patients. Those with a baseline FVC < 65% predicted (*n* = 13) had significantly shorter length of treatment prior to discontinuation of therapy due to ADRs compared to those with FVC % predicted 65–80% (*n* = 16) (^∗^*p* < 0.05). There was no significant difference in duration of therapy when stratified according to baseline TLCO % predicted. Data presented as means with standard deviation. Statistical analysis: analysis of variance with *post hoc* Holm’s–Sidak comparisons analysis. FVC, forced vital capacity; NICE criteria, National Institute for Health and Care excellence criteria for stopping medication (National Institute for Health and Care Excellence, 2013) (anti-fibrotic medication has to be stopped where there is disease progression as evidenced by a decline in percent predicted FVC of 10% or more within any 12 months period).

The median duration of therapy prior to discontinuation because of ADRs, was not statistically different between groups [204 days pirfenidone versus 122 days nintedanib, ratio of 1.67 (95% CI 0.78 to 3.57)] (Supplementary Figure S2). However, when stratified according to baseline lung function, patients commencing pirfenidone with FVC < 65% predicted, had a significantly shorter duration of therapy prior to discontinuation due to ADRs, compared to those with a FVC 65–80% predicted (Figure 4). We were unable to perform meaningful comparisons for nintedanib, owing to the small patient numbers in this subgroup.

During the follow-up period, there was a total of 56 ADRs in 46 patients, directly contributing to discontinuation of pirfenidone therapy, with most patients reporting one (35%, 16/46) or two ADRs (33%, 15/46). Anorexia (11 ADRs), rash (11 ADRs) and GI disturbance (10 ADRs) were reported most commonly as the reason for cessation of therapy (Figure 2). It was not possible to ascertain those patients who had dose reductions or temporary interruption of treatment in an attempt to mitigate against these ADRs. Over half of those patients eventually terminating pirfenidone due to ADRs chose to switch onto nintedanib therapy (*n* = 26).

In the nintedanib group, there were 9 recorded ADRs resulting in discontinuation of therapy in 6 patients (Figure 3). ADRs leading to termination of therapy in a further 2 patients were not specified. Similarly as for the pirfenidone cohort, it was not possible to ascertain those patients who had dose reductions or temporary interruption of treatment in an attempt to mitigate against these ADRs. In this small cohort, ADRs of anorexia (3 ADRs), nausea (2 ADRs), and weight loss (2 ADRs) were most commonly reported.

### Overall Safety of Anti-fibrotic Medications

There were no deaths considered to be directly related to anti-fibrotic medication. In the 2 cases where liver function derangement occurred at >3 times the upper limit of normal (*n* = 1 for nintedanib, *n* = 1 pirfenidone), biochemistry resolved spontaneously upon cessation of medication. Specifically, there were no cardiac, cerebrovascular or bleeding adverse events in the nintedanib subgroup.

### Treatment-Emergent Adverse Events of Anti-fibrotic Medication at 52 Weeks Follow-Up

We then examined the treatment emergent adverse events for both pirfenidone and nintedanib at 52 weeks of follow-up (and additionally at 104 weeks for pirfenidone), enabling more direct comparison of the tolerability of anti-fibrotic medications with clinical trial data.

**FIGURE 5:**
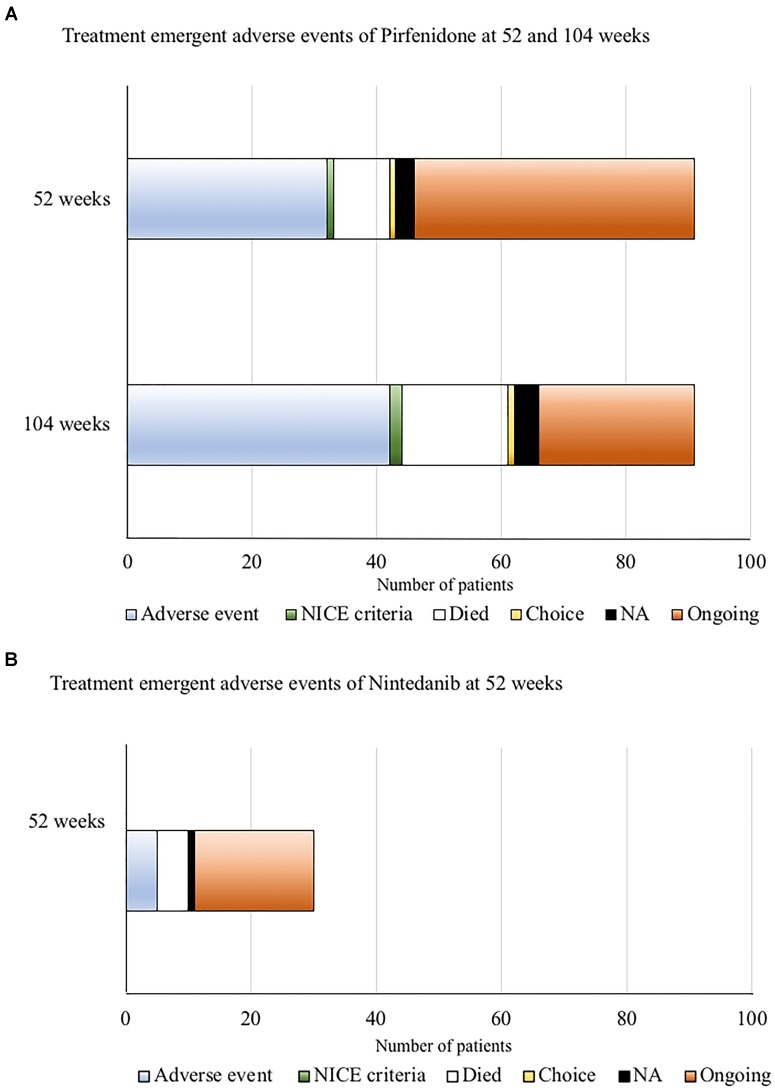
**(A)** Treatment emergent adverse events for pirfenidone at 52 and 104 weeks of follow-up. Only patients with complete data sets at both time points were included (*n* = 91). Of these, 35% (32/91) had discontinued treatment within the 1st year of therapy due to adverse drug reactions (ADRs). A further 11% (10/91) of this cohort discontinued therapy due to ADRs within the 2nd year of therapy. Nine patients died within the 1st year (10%, 9/91), and a further 8 patients during the 2nd year of follow up (equating to 19%, 17/91 by the end of the 2nd year of follow up). Therapy was stopped in two patients (1 by 52 weeks and 1 by 104 weeks of follow-up) according to National Institute for Health and Care excellence (NICE) criteria for stopping medication (National Institute for Health and Care Excellence, 2013) (anti-fibrotic medication has to be stopped where there is disease progression as evidenced by a decline in percent predicted FVC of 10% or more within any 12 months period). Therefore overall, 46% (42/91) patients treated with 52 weeks of pirfenidone had a TEAE that directly led to discontinuation of therapy (ADRs *n* = 32, death *n* = 9, disease progression *n* = 1). **(B)** Treatment emergent adverse events for nintedanib at 52 weeks of follow up. Of those patients with follow-up data at 52 weeks of nintedanib (*n* = 30), 16.7% (5/30) discontinued therapy due to ADRs, all occurring within the first 6 months of follow-up. Five patients died. The reason for cessation of therapy in one patient could not be determined. Overall, 33% (11/30) patients had TEAEs (ADRs *n* = 5, death *n* = 5, disease progression *n* = 0, unknown *n* = 1) that directly led to discontinuation of nintedanib therapy at 52 weeks.

Of those patients with complete follow-up data at 52 and 104 weeks following commencement of pirfenidone (*n* = 91) (Figure 5A), 35% (32/91) discontinued treatment within the 1st year of therapy due to ADRs, with over half of these (56%, 18/32) occurring within the first 6 months. A further 11% (10/91) of this cohort discontinued therapy due to ADRs within the 2nd year of therapy. Nine patients died within the 1st year (10%, 9/91), and a further 8 patients during the 2nd year of follow up (equating to 19%, 17/91 by the end of the 2nd year of follow up). Therapy was stopped in two patients (1 by 52 weeks and 1 by 104 weeks of follow-up) due to evidence of disease progression. Therefore, 46% (42/91) patients treated with 52 weeks of pirfenidone had a TEAE that directly led to discontinuation of therapy (ADRs *n* = 32, death *n* = 9, disease progression *n* = 1).

**FIGURE 6:**
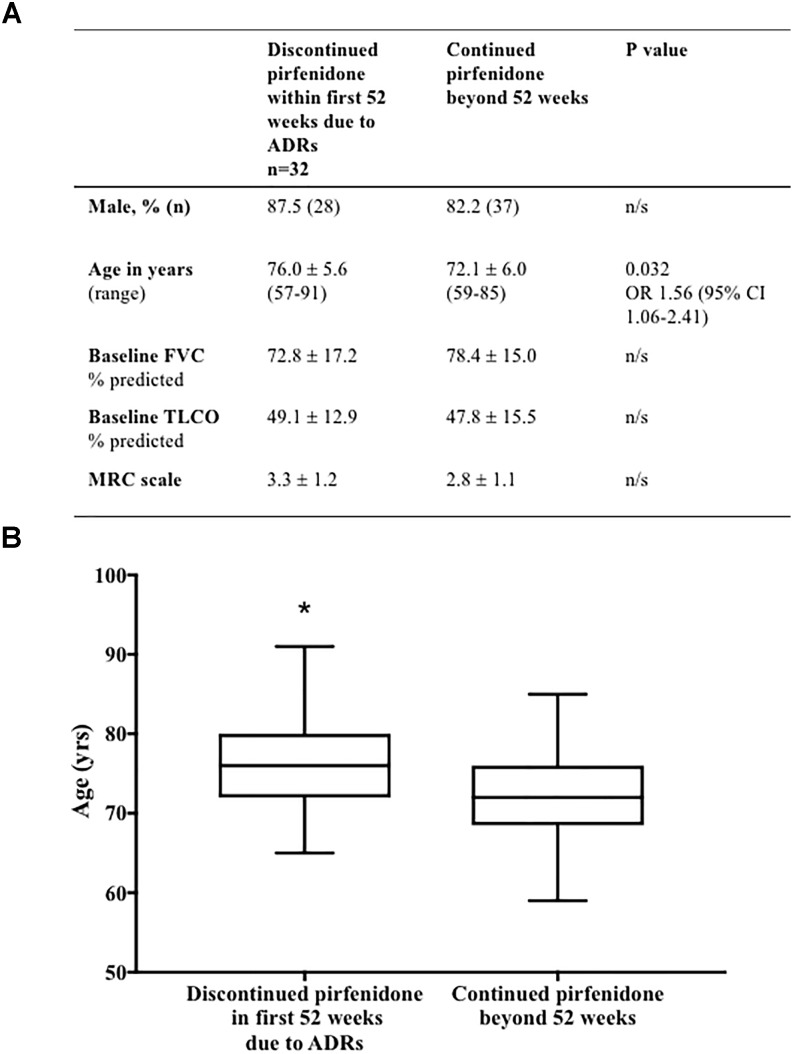
**(A)** Baseline demographics associated with discontinuation of pirfenidone due to adverse drug reactions. Comparison of baseline patient characteristics (age, gender, baseline FVC % predicted, TLCO % predicted and MRC scale) of patients who discontinued pirfenidone within the 1st year of treatment, as a result of ADRs, compared to those who continued therapy beyond 52 weeks of follow up. Univariable logistic regression identified older age to be associated with discontinuation (^∗^*p* = 0.032) [odds ratio (OR)] of discontinuing treatment 1.56 times higher, for every 5 years increase in age (95% CI 1.06–2.41) (Graphically represented in **B**). There was no statistical association between gender, baseline FVC % predicted, TLCO % predicted or MRC scale and discontinuation of therapy. Results presented as mean ± SD unless otherwise stated. Analysis excludes those patients without complete data sets (*n* = 4), and those terminating therapy within the first 52 weeks due to disease progression (*n* = 9) or death (*n* = 1). FVC, forced vital capacity; n, number; FVC, forced vital capacity; TLCO, transfer factor for carbon monoxide; MRC, medical research council; CI, confidence interval.

Comparison of baseline patient characteristics (age, gender, baseline FVC % predicted, TLCO % predicted and MRC scale) of patients who discontinued pirfenidone within the 1st year of treatment, as a result of ADRs, compared to those who continued therapy beyond 52 weeks of follow up, identified older age to be associated with discontinuation (*p* = 0.032) [odds ratio of discontinuing treatment 1.56 times higher, for every 5 years increase in age (95% CI 1.06–2.41)] (Figure 6).

Multivariable logistic regression analysis of these baseline patient demographics (age, gender, baseline FVC % predicted, TLCO % predicted and MRC scale) suggested that increasing age was not an independent predictor of discontinuation of pirfenidone due to ADRs at the 5% statistical significance level (*p* = 0.093).

Of those patients with follow-up data at 52 weeks of nintedanib (Figure 5B), 17% (5/30) of patients discontinued therapy within the 1st year due to ADRs, all occurring within the first 6 months of follow-up. Overall, 33% (11/30) patients had TEAEs (ADRs *n* = 5, death *n* = 5, disease progression *n* = 0, unknown *n* = 1) that directly led to discontinuation of nintedanib therapy. Baseline characteristics predicting discontinuation of nintedanib treatment was not examined, due to the small patient numbers.

## Discussion

Two anti-fibrotic therapies are currently approved for the treatment of IPF based on the results of large scale phase III clinical trials (Noble et al., 2011; King et al., 2014; Richeldi et al., 2014). Despite the existence of these large scale Phase III clinical trials and open label follow-on studies (Koschel et al., 2014; Crestani et al., 2016; Costabel et al., 2017) value can be gained from the documentation of clinical experiences with anti-fibrotics in patient populations that may not fit the stringent criteria required for clinical trial inclusion. This is particularly true for nintedanib that was only licensed for European use in 2015.

This study evaluated the safety and drug tolerability of pirfenidone and nintedanib for the treatment of IPF in a tertiary ILD referral center. We present results from a group of patients with moderate IPF, an IPF population representative of those currently licensed to receive anti-fibrotics in the United Kingdom. Low numbers of females received anti-fibrotic treatment in our cohort, a demographic that is consistent with large scale epidemiological studies of IPF (Navaratnam et al., 2011; Snell et al., 2016). Both pirfenidone and nintedanib were discontinued most commonly due to ADRs. No new safety concerns were identified.

In the pooled data from CAPACITY/ASCEND (Noble et al., 2016), TEAEs related to pirfenidone therapy were generally mild to moderate in severity. Study treatment was discontinued prematurely in 19.8% (55/27), a figure that included ADRs, deaths on therapy and disease progression during the follow up period. This is lower than reported in our own cohort (46% at 52 weeks), but comparable to that described in RECAP (TEAEs leading to treatment discontinuation in 42% patients over mean of 122 weeks) (Costabel et al., 2017) and real-world datasets such as Oltmanns et al. (2014) who described TEAEs leading to treatment discontinuation in 39% patients with mean follow-up of 11-months (with ADRs of 20%, deaths in 11% and disease progression in 8%) in a single-center German cohort.

Gastrointestinal symptoms, including nausea, anorexia and weight loss, and skin-related symptoms including photosensitivity, were most commonly reported as the ADRs of pirfenidone in our cohort, consistent with clinical trial data (Noble et al., 2011; King et al., 2014; Valeyre et al., 2014).

When patients who had discontinued pirfenidone due to ADRs were stratified according to baseline lung function, those with FVC < 65% predicted, had a significantly shorter duration of pirfenidone therapy prior to discontinuation compared to those with a FVC 65–80% predicted. It is possible that those with more severe disease at presentation are less tolerant of treatment related side effects. This contrasts with clinical trial data (Noble et al., 2011; King et al., 2014) and the post-marketing surveillance of pirfenidone in Japanese patients with IPF (Ogura et al., 2015) and may be explained, in part, by our significantly older population. Increasing age was also associated with discontinuation of pirfenidone because of ADRs and prior to 52 weeks follow-up (odds ratio of discontinuing treatment 1.56 times higher, for every 5 years increase in age), although there were no independent predictors identified on multivariate analysis. A recently published retrospective analysis of the long term efficacy and safety of pirfenidone in Japan (Ogawa et al., 2018) identified that on multivariate analysis, an % FVC < 60% was an independent predictor of the inability to take pirfenidone for over a year. Furthermore, Tzouvelekis et al. (2017) investigated the safety and efficacy of pirfenidone therapy in 43 patients with severe lung function impairment (% FVC < 50% and %DLCO < 35%) and identified treatment discontinuation due to adverse events occurred in 20% patients, slightly higher than described in CAPACITY (Noble et al., 2011) and ASCEND (King et al., 2014). Harari et al. (2018) have provided initial evidence to suggest that nintedanib slows the rate of decline of absolute and %predicted TLCO but not FVC in a retrospective analysis of patients with severe IPF (FVC < 50%), a cohort not currently licensed to receive nintedanib in the United Kingdom. Based on these observations further exploration into the tolerability and effectiveness of anti-fibrotic medications in those with more severe disease is required.

The largest ‘real world’ dataset of early clinical experience of nintedanib in IPF, examined its use via the manufacturer funded ‘patient in need’ scheme, prior to NICE approval (Toellner et al., 2017). Undertaken in the United Kingdom, many of the patients had mild IPF with a mean FVC % predicted of 81.1 (SD 19.8) in the population studied; a value that lies above the current NICE guidelines for commencement of anti-fibrotic treatment, and thus the generalisability of results to the United Kingdom IPF population might be questioned. Very few studies of real world experience of nintedanib have been published since it received a European license in 2015 (Bonella et al., 2016; Galli et al., 2017; Brunnemer et al., 2018; Tzouvelekis et al., 2018). The current study, whilst comparable in size to existing small-scale retrospective studies, has the added benefit of observing outcomes over a longer period in all but one of these studies (Brunnemer et al., 2018) and reassuringly no new safety concerns were identified.

Treatment emergent adverse events leading to discontinuation of nintedanib was 33% at 52-weeks, compared to 25.2% in INPULSIS-1 and 23.7% in INPULSIS-2 phase III clinical trials (Richeldi et al., 2014). Our data is comparable to post-marketing surveillance programs (Noth et al., 2016) and other real-world datasets, although differences in methodology make direct comparisons difficult. A single-center German cohort reported discontinuation rates of 28% (18/64) during a mean follow up of 11 months (Brunnemer et al., 2018) and 39.3% at 52-weeks (21.2% from severe adverse events and 18.1% deaths, *n* = 94) in a multi-center Greek cohort (Tzouvelekis et al., 2018). A multi-center United Kingdom study reported discontinuation rates of 21% (39/187) in a milder IPF patient cohort (average FVC was 81.1% predicted, with more than 50% patients having an FVC > 80% predicted), a value that excluded those patients who had died during the follow-up period, an additional 10% (Toellner et al., 2017).

In accordance with these real-world datasets of nintedanib (Bonella et al., 2016; Galli et al., 2017; Toellner et al., 2017; Brunnemer et al., 2018; Tzouvelekis et al., 2018), gastrointestinal ADRs were most commonly reported in our patients. No bleeding or cardiovascular events were reported, validating previously published phase III clinical trial data (cardiovascular events 2.7%) (Richeldi et al., 2014).

Several approaches are routinely adopted within our center and others (Noth et al., 2016) to avoid or alleviate ADRs related to antifibrotic medications. These include advice on taking medication with food and in divided doses throughout the course of a meal, particularly for gastro-intestinal related ADRs with slowed dose titration (for pirfenidone), treatment breaks and additional loperamide use if patients experience diarrhea. Patients prescribed pirfenidone are advised to use daily high factor sunblock with UVA and UVB protection and to wear protective clothing to help mitigate against skin-related ADRs.

The true incidence of ADRs may have been underestimated in our cohort as we were unable to capture information on which of these different approaches had been attempted by individuals prior to discontinuation of therapy. Furthermore, the data set represents a natural learning curve for the health care professionals within our service on the use of these new anti-fibrotic medications and how best to manage ADRs, with the development of an internal ‘center-threshold,’ based on our experiences, when to advise treatment cessation, something that cannot be controlled for statistically.

In accordance with analysis of large populations of IPF patients receiving anti-fibrotic treatment (Valeyre et al., 2014; Noth et al., 2016), we observed that ADRs leading to discontinuation of therapy were most common in the first 6 months of treatment for both nintedanib and pirfenidone. However, a cohort of pirfenidone patients (11%) reported ADRs causing cessation therapy as late as 2 years after treatment initiation. A new 801 mg tablet formulation of pirfenidone has recently been approved, as a bioequivalent option for patients established on maximum treatment, to reduce pill burden (Cottin and Maher, 2015). This data highlights the potential for delayed ADRs in those already stabilized on therapy and raises uncertainties as to whether this new preparation will reduce flexibility for some patients in controlling these more delayed events.

We acknowledge other potential limitations of this study including those inherent to retrospective observational studies, the absence of appropriate controls and potential bias concerning missing data. The small patient numbers described in this study, particularly of the nintedanib subgroup, is a clear limitation and reflects the later approval of nintedanib for clinical use in the United Kingdom. Accordingly, direct comparisons between the anti-fibrotics medications should be made judiciously.

Finally, in contrast to many of the existing published real world experiences of the use of anti-fibrotic medications in IPF, this current cohort includes patients prescribed pirfenidone or nintedanib as part of a named patient program, mild patient program and treated as per NICE recommendations (National Institute for Health and Care Excellence, 2013). It is unclear as to the psychological influence of these different programs on an individuals’ tolerance of ADRs and indeed whether the potential option of switching to a second line medication impacts on what is deemed an ‘acceptable’ and ‘manageable’ ADRs by both patients and health care professionals.

In summary, patients in our cohort with IPF prescribed nintedanib had comparable TEAE profiles in clinical practice to those reported in clinical trials. The TEAE profile of pirfenidone was higher than clinical trial data would suggest, although comparable to real-world datasets. Further work is required to explore the possible reasons underpinning this finding, including whether this is related to population co-morbidities or center threshold.

## Author Contributions

SB analyzed the data and wrote the first draft of the manuscript. SM drafted the project idea. KA and SM performed the data collection. All other members were involved in the final manuscript preparation.

## Conflict of Interest Statement

The authors declare that the research was conducted in the absence of any commercial or financial relationships that could be construed as a potential conflict of interest.

## Supplementary Material

FIGURE S1 Comparison of baseline demographics of the pirfenidone and nintedanib subgroups with pooled data of the baseline characteristics of patients in the Phase III clinical trials of these medications. **(A)** Patient characteristics of the pirfenidone group compared to the pooled dataset from CAPACITY and ASCEND (Noble et al., 2016). The study group was significantly older and a higher proportion were male compared to the clinical trial data. ˆ Mean and SD of age in years derived from the ASCEND trial information only (King et al., 2014). ∫ Data pooled from ASCEND and CAPACITY (Noble et al., 2016)suggests range in age of 45–80 years, with average of 68.0 years. ^∗^5 patients were unable to perform the gas transfer maneuver due to cough. **(B)** Patient characteristics of the nintedanib group compared to the pooled dataset from INPULSIS 1+2^15^. n, number; FVC, forced vital capacity; TLCO, transfer factor for carbon monoxide; S.D., standard deviation. *P*-value calculated statistical significance value; ns, non-significant statistically. Data presented as means with standard deviation. Statistical analysis: analysis of variance with *post hoc* Holm–Sidak multiple comparisons analysis.

FIGURE S2 Kaplan–Meier curve analysis determining the median duration of pirfenidone and nintedanib therapy in days, prior to discontinuation due to adverse drug reactions (ADRs). Complete data was available for 97 pirfenidone patients and 47 nintedanib patients. There was no significant difference in the duration of nintedanib or pirfenidone therapy prior to discontinuation (median therapy 204 days pirfenidone versus 122 days nintedanib, ratio of 1.67 (95% CI 0.78–3.57) *p* = 0.707 Log-rank (Mantel-Cox) test. Statistical analysis: analysis of variance with *post hoc* Holm’s–Sidak multiple comparisons analysis.
