# Supplementary figures and images for: South–West of England’s Experience of the Safety and Tolerability Pirfenidone and Nintedanib for the Treatment of Idiopathic Pulmonary Fibrosis (IPF)
Source: Front Pharmacol. 2018 Dec 17;9:1480. doi: 10.3389/fphar.2018.01480 (PMC6304353; doi:10.3389/fphar.2018.01480)

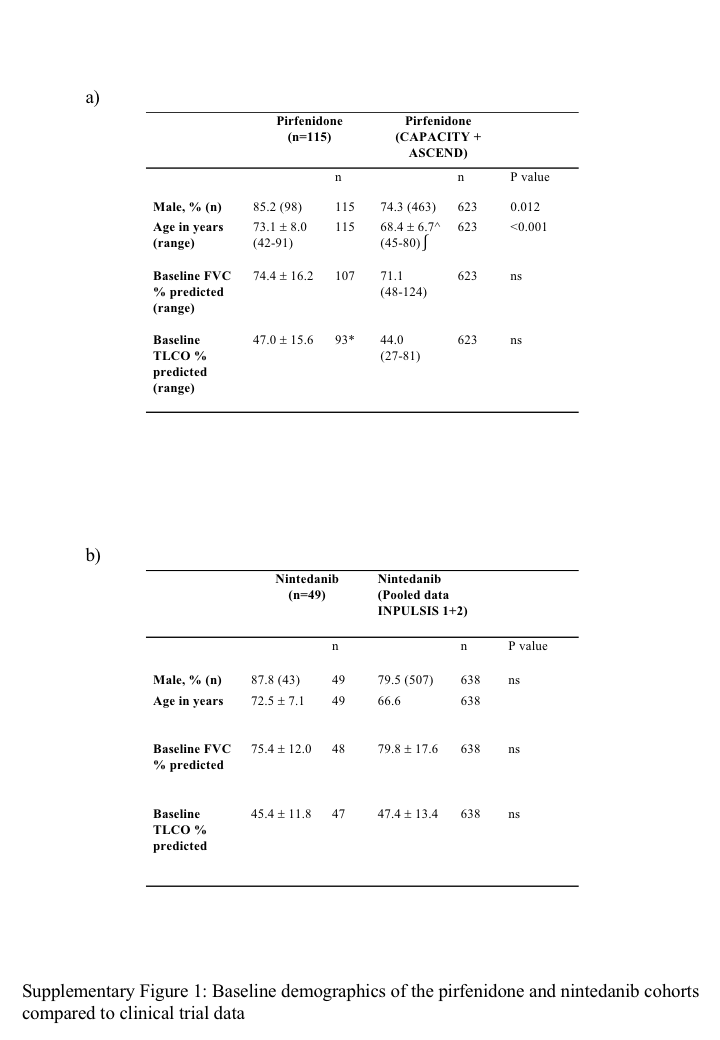

Supplement: FIGURE S1 — Comparison of baseline demographics of the pirfenidone and nintedanib subgroups with pooled data of the baseline characteristics of patients in the Phase III clinical trials of these medications. (A) Patient characteristics of the pirfenidone group compared to the pooled dataset from CAPACITY and ASCEND (Noble et al., 2016). The study group was significantly older and a higher proportion were male compared to the clinical trial data. ˆ Mean and SD of age in years derived from the ASCEND trial information only (King et al., 2014). ∫ Data pooled from ASCEND and CAPACITY (Noble et al., 2016)suggests range in age of 45–80 years, with average of 68.0 years. ∗5 patients were unable to perform the gas transfer maneuver due to cough. (B) Patient characteristics of the nintedanib group compared to the pooled dataset from INPULSIS 1+215. n, number; FVC, forced vital capacity; TLCO, transfer factor for carbon monoxide; S.D., standard deviation. P-value calculated statistical significance value; ns, non-significant statistically. Data presented as means with standard deviation. Statistical analysis: analysis of variance with post hoc Holm–Sidak multiple comparisons analysis. [file Image_1.tiff]

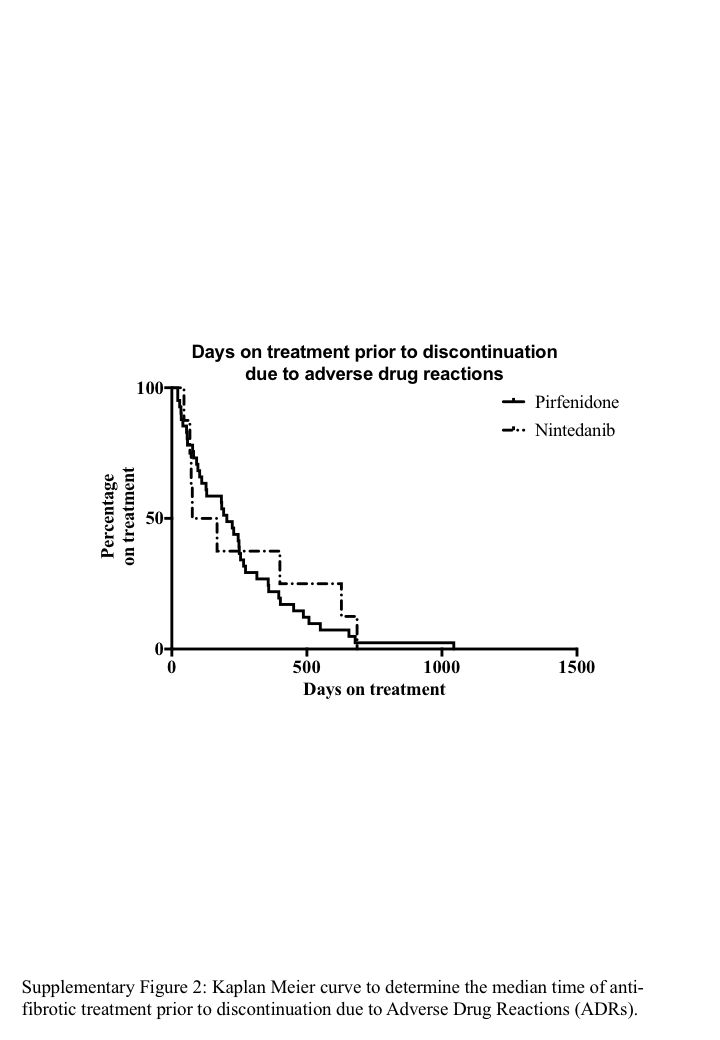

Supplement: FIGURE S2 — Kaplan–Meier curve analysis determining the median duration of pirfenidone and nintedanib therapy in days, prior to discontinuation due to adverse drug reactions (ADRs). Complete data was available for 97 pirfenidone patients and 47 nintedanib patients. There was no significant difference in the duration of nintedanib or pirfenidone therapy prior to discontinuation (median therapy 204 days pirfenidone versus 122 days nintedanib, ratio of 1.67 (95% CI 0.78–3.57) p = 0.707 Log-rank (Mantel-Cox) test. Statistical analysis: analysis of variance with post hoc Holm’s–Sidak multiple comparisons analysis. [file Image_2.TIFF]
